# Supplementary material for: The evolving systemic biomarker milieu in obese ZSF1 rat model of human cardiometabolic syndrome: Characterization of the model and cardioprotective effect of GDF15
Source: PLoS One. 2020 Aug 17;15(8):e0231234. doi: 10.1371/journal.pone.0231234 (PMC7430742; doi:10.1371/journal.pone.0231234)
Supplement: S1 File — (DOCX) [file pone.0231234.s001.docx]

**Materials and methods**

**Gene models used for alignment and quantification**

RNA-seq alignment and quantification utilized slightly modified versions of the gene model, genome build 38 using GENCODE v24 annotation, curated by OmicSoft (Cary, NC, USA). The modifications to the gene models imposed a more stringent definition of the term “gene,” requiring that all of the gene’s transcripts reside on the same chromosome and strand, and that the distance between a gene’s transcripts not span more than 10 kilobases that is not covered by any (unspliced) transcript. Genes that violated this definition were split into multiple genes to comply with these criteria, and the gene names were appended with a suffix indicating the strand, chromosome name, and transcript bundle number.

**Read filtering and trimming**

All read files were pre-processed to remove poor quality reads and to trim poor quality 3′ bases. First, any read pair containing a read that failed the Illumina quality filter was removed. Next, all reads were trimmed from the 3′ terminus until either (a) the read length was reduced to 40 bp or (b) less than two of the bases in the five-base window at the 3′ terminus of the read had a quality score value below 3. This trimming included a final polishing step in which the base at the 3′ terminus was trimmed if the read length exceeded 40 bp and the quality score of the base was below 3.

**Alignment and quantification**

The filtered and trimmed reads were aligned and quantified using OmicSoft’s Array Studio software (Oshell.exe v10.0) [1]. This software compared favourably to other commonly used tools (e.g., Tophat/Cufflinks, MMSEQ) in internal benchmark comparisons. The alignment algorithm is documented in an OmicSoft whitepaper [2], and the gene/transcript quantification module is an OmicSoft implementation of the RSEM algorithm [3].

**Differential expression analysis**

Genes with critical issues on gene models, including duplicated regions, high GC content, and overlapping transcripts were removed from statistical analysis. Genes expressed at fragments per kilobase per million quantiles normalized (FPKQ) ≥ 1 in at least five samples were selected for further statistical test. Differential expression analysis was performed using DESeq2 v1.18.1 [4]. Raw read counts from the selected genes were compared using R Bioconductor package DESeq2 following negative binomial distribution. Gene expression fold changes were calculated using FPKQ values. Genes with Benjamini-Hochberg (BH) corrected *p-*value < 0.05 and fold change ≥ 1.5 or ≤ 2/3 were selected as significantly differentially expressed genes.

**Expression normalization**

The gene- and transcript-level expression of each RNA-seq sample was reported by Oshell as a fragments per kilobase per million sequenced (FPKM) value. FPKM is an expression measurement based on the count of library fragments that map to a given feature (gene or transcript) normalized for feature length and the number of fragments sequenced. These values were adjusted on a per-sample basis using 70th percentile normalization, yielding a final expression metric which we refer to as “FPKQ”.

**References**

1. OmicSoft. [www.omicsoft.com/array-studio.php](https://amgen-my.sharepoint.com/personal/ccarte02_amgen_com/Documents/Obesity%20Pipeline/171%20Pubs/Manuscripts/Biomarker%20Research/www.omicsoft.com/array-studio.php). Accessed 20 January 2020.

2. OmicSoft. OmicSoft Aligner. [www.omicsoft.com/downloads/whitepaper/OmicsoftAligner.pdf](https://amgen-my.sharepoint.com/personal/ccarte02_amgen_com/Documents/Obesity%20Pipeline/171%20Pubs/Manuscripts/Biomarker%20Research/www.omicsoft.com/downloads/whitepaper/OmicsoftAligner.pdf). Accessed 20 January 2020.

3. Li B, Dewey CN. RSEM: accurate transcript quantification from RNA-Seq data with or without a reference genome. BMC Bioinformatics. 2011;12:323.

4. Love MI, Huber W, Anders S. Moderated estimation of fold change and dispersion for RNA-seq data with DESeq2. Genome Biol. 2014;15(12):550.
