# Supplementary material for: The evolving systemic biomarker milieu in obese ZSF1 rat model of human cardiometabolic syndrome: Characterization of the model and cardioprotective effect of GDF15
Source: PLoS One. 2020 Aug 17;15(8):e0231234. doi: 10.1371/journal.pone.0231234 (PMC7430742; doi:10.1371/journal.pone.0231234)
Supplement: S1 Table — (DOCX) [file pone.0231234.s002.docx]

**Supplementary Table 1.** Metabolic and renal biomarkers in serum/plasma of 20-week-old lean and obese ZSF1 male rats.

| **Biomarker** | **Serum concentration (mean ± SEM)** | | ***p-*value** |
| --- | --- | --- | --- |
|  | **Lean ZSF1** | **Obese ZSF1** |  |
| **Cholesterol (mg/dL)** | **64.6 ± 2.0** | **271.1 ± 13.4** | ***<0.0001*** |
| **Triglycerides (mg/dL)** | **118.5 ± 7.6** | **3239 ± 163** | ***<0.0001*** |
| **BUN (mg/dL)** | **20.5 ± 0.5** | **23.5 ± 1.0** | ***0.0178*** |
| LDL (mg/dL) | 11.2 ± 0.3 | 12.0 ± 0.6 | 0.2749 |
| **Glucose (mg/dL)** | **110.0 ± 3.6** | **350.6 ± 29.4** | ***<0.0001*** |
| **Insulin (ng/mL)** | **2.9 ± 0.6** | **11.1 ± 2.9** | ***0.0164*** |
| **Pro-insulin (pmol/L)** | **27.8 ± 3.9** | **1337 ± 130** | ***<0.0001*** |
| **C-peptide (ng/mL)** | **2.4 ± 0.2** | **6.4 ± 0.4** | ***<0.0001*** |
| **Amylin (active, pg/mL)** | **45.6 ± 8.1** | **288.6 ± 25.6** | ***<0.0001*** |
| **Leptin (ng/mL)** | **6.8 ± 0.9** | **41.0 ± 6.3** | ***<0.0001*** |
| GIP (pg/mL) | 223.1 ± 20.0 | 218.6 ± 51.3 | 0.9372 |
| **Glucagon (pg/mL)** | **25.8 ± 2.5** | **37.8 ± 4.6** | ***0.0388*** |
| PP (pg/mL) | 26.1± 3.9 | 34.6 ± 8.0 | 0.3599 |
| **PYY (pg/mL)** | **32.8 ± 3.0** | **106.3 ± 13.8** | ***<0.0001*** |
| **Adiponectin (µg/mL)** | **2.58 ± 0.09** | **1.85 ± 0.05** | ***<0.0001*** |
| **Clusterin (µg/mL)** | **8.6 ± 0.2** | **12.0 ± 0.4** | ***<0.0001*** |
| **KIM-1 (pg/mL)** | **294.0 ± 7.0** | **641.8 ± 94** | ***0.0024*** |
| **NGAL (ng/mL)** | **43.7 ± 2.4** | **66.1 ± 4.3** | ***0.0005*** |
| MCP1 (ng/mL) | 1.19 ± 0.09 | 1.28 ± 0.12 | 0.1497 |
| **TNFa (pg/mL)** | **29.4 ± 3.4** | **12.6 ± 2.9** | ***0.0022*** |
| **IL-1b (pg/mL)** | **39.1 ± 6.6** | **121.4 ± 26.7** | ***0.0098*** |
| TGFb (ng/mL) | 75.1 ± 1.5 | 70.0 ± 3.4 | 0.1934 |
| TIMP1 (ng/mL) | 3.33 ± 0.47 | 4.21 ± 0.34 | 0.1524 |
