# Supplementary material for: The evolving systemic biomarker milieu in obese ZSF1 rat model of human cardiometabolic syndrome: Characterization of the model and cardioprotective effect of GDF15
Source: PLoS One. 2020 Aug 17;15(8):e0231234. doi: 10.1371/journal.pone.0231234 (PMC7430742; doi:10.1371/journal.pone.0231234)
Supplement: S2 Table — (DOCX) [file pone.0231234.s003.docx]

**Supplementary Table 2.** Echocardiographic and exercise capacity parameters in lean and obese ZSF1 female rats.

| **Biomarker** | **Physical parameters (mean ± SEM)** | | ***p-*value** |
| --- | --- | --- | --- |
|  | **Lean ZSF1**  **(*n* = 8–12)** | **Obese ZSF1**  **(*n* = 8–12)** |  |
| **Body weight (g)** | **246 ± 2** | **442 ± 4** | ***<0.0001*** |
| **Heart rate (bpm)** | **375 ± 7** | **348 ± 9** | ***0.025*** |
| Diameter (s) (mm) | 3.4 ± 0.1 | 3.2 ± 0.2 | 0.5 |
| Diameter (d) (mm) | 7.1 ± 0.1 | 7.3 ± 0.2 | 0.37 |
| Volume (s) (µL) | 49 ± 5 | 46 ± 7 | 0.68 |
| Volume (d) (µL) | 265 ± 9 | 282 ± 14 | 0.31 |
| **Stroke volume (µL)** | **216 ± 5** | **236 ± 8** | ***0.04*** |
| Ejection fraction (%) | 82 ± 1 | 85 ± 2 | 0.23 |
| Fractional shortening (%) | 53 ± 1 | 57 ± 3 | 0.17 |
| Cardiac output (mL/min) | 81 ± 1 | 82 ± 3 | 0.7 |
| **LV mass (mg)** | **1050 ± 31** | **1934 ± 155** | ***0.00001*** |
| **LV mass cor (mg)** | **840 ± 25** | **1547 ± 124** | ***0.00001*** |
| **IVRT (ms)** | **18.3 ± 0.4** | **22.8 ± 0.8** | ***0.00003*** |
| E/E’ (ratio) | 15.5 ± 1.8 | 16.5 ± 0.7 | 0.626 |
| **Distance (m)** | **386 ± 136** | **221 ± 24** | ***0.0025*** |
| **Time to exhaustion (min)** | **24 ± 1** | **17 ± 1** | ***0.0004*** |
| **Peak VO_2_ (mg/kg/h)** | **3921 ± 177** | **3020 ± 139** | ***0.001*** |
| Respiratory exchange (ratio) | 0.89 ± 0.03 | 0.83 ± 0.03 | 0.186 |
