# Supplementary material for: The evolving systemic biomarker milieu in obese ZSF1 rat model of human cardiometabolic syndrome: Characterization of the model and cardioprotective effect of GDF15
Source: PLoS One. 2020 Aug 17;15(8):e0231234. doi: 10.1371/journal.pone.0231234 (PMC7430742; doi:10.1371/journal.pone.0231234)
Supplement: S3 Table — (DOCX) [file pone.0231234.s004.docx]

**Supplementary Table 3.** Cardiovascular biomarkers in circulation of 20-week-old lean and obese ZSF1 male rats.

| **Biomarker** | **Serum/plasma concentration (mean ± SEM)** | | ***p-*value** |
| --- | --- | --- | --- |
|  | **Lean ZSF1** | **OB ZSF1** |  |
| Follistatin-like 1 (ng/mL) | **8.11 ± 0.18** | **6.92 ± 0.17** | ***0.0002*** |
| Osteopontin (ng/mL) | 0.96 ± 0.02 | 0.96 ± 0.03 | 0.9845 |
| **GDF15 (pg/mL)** | **62.0± 1.8** | **161.9 ± 17.3** | ***<0.0001*** |
| BNP (pg/mL) | 27.1 ± 3.4 | 34.7 ± 5.4 | 0.2512 |
| sE-selectin (ng/mL) | 98.6 ± 5.4 | 93.8 ± 3.4 | 0.4497 |
| **FABP3 (pg/mL)** | **64.8 ± 6.15** | **185.9 ± 32.36** | ***0.0025*** |
| FABP4 (ng/mL) | 42.41 ± 7.34 | 56.54 ± 7.63 | 0.2030 |
| **FABP5 (ng/mL)** | **11.28 ± 0.52** | **18.84 ± 1.16** | ***<0.0001*** |
| **Aldosterone (ng/mL)** | **0.708 ± 0.081** | **1.658 ± 0.223** | ***0.0010*** |
| NT-proANP (ng/mL) | 9.67 ± 0.75 | 9.17 ± 0.92 | 0.6768 |
| **NT-proBNP (pg/mL)** | **99.18 ± 12.55** | **34.91 ± 3.39** | ***0.0002*** |
| MYBPC3 | 15.32 ± 2.85 | 9.12 ± 1.79 | 0.0864 |
| **IL-16 (pg/mL)** | **55.4 ± 2.26** | **418.3 ± 50.3** | ***<0.0001*** |
| **ST2 (pg/mL)** | **435.9 ± 18.8** | **711.7 ± 29.4** | ***<0.0001*** |
| Endothelin-1 (ET1) (pg/mL) | 1.48 ± 0.09 | 1.9 ± 0.19 | 0.0686 |
| FLT1/VEGFR1 (pg/mL) | 16.9 ± 1.22 | 19.32 ± 2.15 | 0.3447 |
