# Supplementary material for: The evolving systemic biomarker milieu in obese ZSF1 rat model of human cardiometabolic syndrome: Characterization of the model and cardioprotective effect of GDF15
Source: PLoS One. 2020 Aug 17;15(8):e0231234. doi: 10.1371/journal.pone.0231234 (PMC7430742; doi:10.1371/journal.pone.0231234)
Supplement: S4 Table — (DOCX) [file pone.0231234.s005.docx]

**Supplementary Table 4.** Effect of 12-week–long Fc-hGDF15 treatment on systemic levels of cardiovascular markers.

| **Biomarker** | **Serum concentration at week 12 of Fc‑hGDF15 treatment (mean ± SEM)** | | ***p-*value** |
| --- | --- | --- | --- |
|  | **Vehicle** | **Fc-hGDF15** |  |
| **Adiponectin (µg/mL)** | **3.15 ± 0.15** | **3.95 ± 0.17** | ***0.0015*** |
| Aldosterone (pg/mL) | 261.5 ± 45.3 | 237.1 ± 45.8 | 0.7118 |
| BNP (pg/mL) | 4.14 ± 1.16 | 4.67 ± 1.66 | 0.8204 |
| **sE-selectin (ng/mL)** | **245.6 ± 12.6** | **146.0 ± 12.0** | ***<0.0001*** |
| FABP3 (ng/mL) | 7.52 ± 1.53 | 5.93 ± 2.28 | 0.5549 |
| **sICAM (ng/mL)** | **29.97 ± 2.01** | **15.72 ± 2.04** | ***<0.0001*** |
| MCP1 (ng/mL) | 4.16 ± 0.24 | 3.52± 0.22 | 0.0683 |
| **Myl3 (pg/mL)** | **154.1 ± 14.41** | **99.9 ± 22.6** | ***0.0458*** |
| NT-proANP (ng/mL) | 20.44 ± 1.30 | 18.08 ± 1.54 | 0.2490 |
| NT-proBNP (pg/mL) | 12.65 ± 1.98 | 15.90 ± 2.53 | 0.3148 |
| **Osteopontin (ng/mL)** | **2.09 ± 0.14** | **1.70 ± 0.12** | ***0.0494*** |
| **TIMP1 (ng/mL)** | **21.04 ± 2.14** | **12.91 ± 2.08** | ***0.01129*** |
| **VEGF (pg/mL)** | **72.87 ± 6.76** | **25.0 ± 10.63** | ***0.0006*** |
| **vWF (ng/mL)** | **186.9 ± 9.83** | **125.1 ± 15.2** | ***0.0016*** |
| GDF15 (pg/mL) | 392.6 ± 33.3 | 443.2 ± 84.2 | 0.5512 |
