# Supplementary material for: The evolving systemic biomarker milieu in obese ZSF1 rat model of human cardiometabolic syndrome: Characterization of the model and cardioprotective effect of GDF15
Source: PLoS One. 2020 Aug 17;15(8):e0231234. doi: 10.1371/journal.pone.0231234 (PMC7430742; doi:10.1371/journal.pone.0231234)
Supplement: S5 Table — (DOCX) [file pone.0231234.s006.docx]

**Supplementary Table 5.** Effect of 12-week–long Fc-hGDF15 treatment on parameters of invasive hemodynamic assessment, echocardiography, and exercise capacity of obese ZSF1 male rats.

| **Biomarker** | **Physical parameters (mean ± SEM)** | | ***p-*value** |
| --- | --- | --- | --- |
|  | **Vehicle** | **Fc-hGDF15** |  |
| **Heart/brain weight (ratio)** | **0.907 ± 0.01** | **0.765 ± 0.01** | ***<0.0001*** |
| Heart rate (bpm) | 294.0 ± 5.8 | 284.9 ± 6.4 | 0.3024 |
| Diameter (s) (mm) | 5.35 ± 0.23 | 5.63 ± 0.13 | 0.3458 |
| Diameter (d) (mm) | 9.56 ± 0.15 | 9.15 ± 0.16 | 0.0711 |
| Volume (s) (µL) | 143.7 ± 14.06 | 156.4 ± 8.42 | 0.4722 |
| Volume (d) (µL) | 512.8 ± 17.93 | 467.8 ± 17.87 | 0.0914 |
| **Stroke volume** **(µL)** | **369.1 ± 10.04** | **311.4 ± 13.02** | ***0.0015*** |
| **Ejection fraction (%)** | **72.59 ± 1.98** | **66.58 ± 1.23** | ***0.0225*** |
| **Fractional shortening (%)** | **44.17 ± 1.75** | **38.63 ± 1.01** | ***0.0167*** |
| **Cardiac output (mL/min)** | **108.7 ± 3.95** | **88.49 ± 3.84** | ***0.0013*** |
| **LV mass (mg)** | **1657 ± 51.83** | **1317 ± 62.41** | ***0.0003*** |
| **LV mass cor (mg)** | **1326 ± 41.46** | **1054 ± 49.93** | ***0.0003*** |
| **IVRT (ms)** | **25.72 ± 0.65** | **28.52 ± 1.19** | ***0.0399*** |
| EDP (mmHg) | 15.94 ± 0.63 | 16.27± 1.02 | 0.7733 |
| Tau (ms) | 13.79 ± 0.57 | 13.86 ± 0.87 | 0.9414 |
| E/E’ (Ratio) | 18.49 ± 0.75 | 16.98 ± 0.73 | 0.1697 |
| **Distance (m)** | **114.9 ± 7.95** | **151.8 ± 12.32** | ***0.0173*** |
| **Time to exhaustion (min)** | **9.98 ± 0.57** | **12.37 ± 0.7** | ***0.0160*** |
| Peak VO_2_ (mg/kg/h) | 3093 ± 74.3 | 3122 ± 123.2 | 0.8331 |
| Respiratory exchange (ratio) | 1.008 ± 0.018 | 0.9949 ± 0.015 | 0.5998 |
