# Supplementary material for: The evolving systemic biomarker milieu in obese ZSF1 rat model of human cardiometabolic syndrome: Characterization of the model and cardioprotective effect of GDF15
Source: PLoS One. 2020 Aug 17;15(8):e0231234. doi: 10.1371/journal.pone.0231234 (PMC7430742; doi:10.1371/journal.pone.0231234)
Supplement: S6 Table — (DOCX) [file pone.0231234.s007.docx]

**Supplementary Table 6.** Heart abundant tissue gene expression increase in LV of obese vs lean ZSF1 groups.

| **Gene** | **Log_2_ FC Increase** | **BH**  ***p*-value** |  | **Gene** | **Log_2_ FC Increase** | **BH**  ***p*-value** |
| --- | --- | --- | --- | --- | --- | --- |
| Acaa2 | 0.925244 | 8.74E–44 |  | Lpar3 | 1.088838 | 1.58E–21 |
| Acadm | 0.623386 | 2.23E–21 |  | Lrp4 | 0.746815 | 5.26E–21 |
| Acot2 | 1.407497 | 5.41E–60 |  | Myh7 | 1.387513 | 3.22E–14 |
| Acox1 | 0.612524 | 2.40E–38 |  | Ncam1 | 1.067203 | 7.08E–11 |
| Adra1a | 0.71756 | 0.000101 |  | Nppa | 0.713072 | 0.0066 |
| Alpk2 | 0.626377 | 3.55E–16 |  | Nrap | 0.713035 | 2.75E–35 |
| Alpk3 | 0.621277 | 9.72E–26 |  | Ntn1 | 0.67961 | 1.05E–14 |
| Ankrd23 | 1.166162 | 2.51E–13 |  | Pank1 | 0.585723 | 3.36E–20 |
| Aqp7 | 0.812687 | 1.77E–18 |  | Pcdh20 | 0.680139 | 0.012131 |
| Cadps | 0.710136 | 2.33E–09 |  | Pcyox1 | 0.601507 | 4.81E–19 |
| Corin | 0.716298 | 1.73E–20 |  | Pde3a | 0.746655 | 2.87E–08 |
| Cpt1a | 0.744796 | 8.84E–18 |  | Pde7b | 0.618594 | 1.51E–05 |
| Csdc2 | 0.616815 | 5.22E–10 |  | Pdk4 | 2.056103 | 2.27E–19 |
| Decr1 | 1.04417 | 2.83E–47 |  | Pdzd2 | 0.664067 | 2.16E–06 |
| Dusp26 | 0.762459 | 9.69E–05 |  | Plekha5 | 0.989596 | 5.11E–15 |
| Ech1 | 0.836496 | 2.03E–37 |  | Postn | 1.02457 | 0.000134 |
| Eci1 | 0.776771 | 3.66E–19 |  | Ppcs | 0.590606 | 3.78E–13 |
| Ehhadh | 0.614765 | 1.37E–10 |  | Ryr2 | 0.586292 | 1.69E–05 |
| Eya4 | 0.645467 | 7.92E–05 |  | Sema6c | 0.940007 | 2.26E–13 |
| Fhl1 | 0.746967 | 0.000126 |  | Slc16a1 | 0.599788 | 8.36E–21 |
| Fkbp5 | 1.020574 | 5.82E–10 |  | Sorbs1 | 0.61142 | 2.33E–06 |
| Gatsl2 | 0.990934 | 1.12E–11 |  | Sorbs2 | 0.691732 | 3.48E–18 |
| Grip2 | 0.813704 | 1.81E–06 |  | Sphkap | 0.948305 | 1.25E–13 |
| Hadha | 0.597629 | 6.57E–22 |  | Synpo | 0.682884 | 5.58E–23 |
| Hadhb | 0.588585 | 7.77E–28 |  | Sypl2 | 1.574139 | 3.43E–11 |
| Hcn4 | 1.787693 | 6.89E–11 |  | Vwa8 | 0.854878 | 9.36E–12 |
| Hipk2 | 1.018523 | 0.000114 |  | Xirp2 | 0.95803 | 3.01E–14 |
| Kcna4 | 0.819119 | 1.56E–08 |  | Zbtb25 | 0.64347 | 5.25E–05 |
