# Supplementary material for: The evolving systemic biomarker milieu in obese ZSF1 rat model of human cardiometabolic syndrome: Characterization of the model and cardioprotective effect of GDF15
Source: PLoS One. 2020 Aug 17;15(8):e0231234. doi: 10.1371/journal.pone.0231234 (PMC7430742; doi:10.1371/journal.pone.0231234)
Supplement: S7 Table — (DOCX) [file pone.0231234.s008.docx]

**Supplementary Table 7.** Heart abundant tissue gene expression decrease in LV of obese vs lean ZSF1 groups.

| **Gene** | **Log_2_ FC Decrease** | **BH**  ***p*-value** |  | **Gene** | **Log_2_ FC Decrease** | **BH**  ***p*-value** |
| --- | --- | --- | --- | --- | --- | --- |
| Ace2 | –0.65349 | 1.51E–07 |  | Mrpl33 | –6.523 | 1.93E–09 |
| Alkbh7 | –0.66233 | 1.11E–07 |  | Myct1 | –0.65596 | 1.04E–06 |
| Apln | –0.98546 | 1.93E–17 |  | Myh6 | –0.83876 | 5.9E–08 |
| Apold1 | –1.29498 | 2.45E–08 |  | Myl2 | –3.4613 | 1.48E–35 |
| Atp1a2 | –0.6179 | 1.36E–17 |  | Myo16 | –0.91175 | 5.52E–05 |
| Atp5i | –1.03852 | 0.000927 |  | Myoz1 | –0.73592 | 0.003804 |
| Aurka | –0.62431 | 0.000256 |  | Ndufa3 | –1.21741 | 0.000483 |
| Btg2 | –1.15314 | 4.11E–08 |  | Nes | –1.04319 | 2.61E–30 |
| C7 | –0.60483 | 0.000167 |  | Ngf | –0.58577 | 0.001143 |
| Cdca7l | –0.66148 | 0.000639 |  | Nr4a2 | –1.2504 | 3.21E–10 |
| Cdh5 | –0.72207 | 3.41E–20 |  | Nrep | –0.94786 | 2.08E–10 |
| Cenpn | –0.6516 | 0.001388 |  | Omd | –0.59348 | 6.99E–05 |
| Cox7a2 | –0.60752 | 1.33E–06 |  | Pcdh12 | –0.9382 | 1.31E–15 |
| Csgalnact1 | –0.60155 | 2.97E–06 |  | Pet100 | –0.59974 | 0.000045 |
| Cxcl1 | –0.97868 | 0.022871 |  | Pfkfb2 | –0.74672 | 5.18E–11 |
| Egr2 | –2.0947 | 2.13E–15 |  | Pomp | –1.989 | 0.000193 |
| Gcat | –0.62848 | 3.12E–05 |  | Slc39a8 | –1.00884 | 5.57E–14 |
| Gimap8 | –0.7251 | 1.45E–12 |  | Slirp | –0.61681 | 1.13E–05 |
| Hspe1 | –0.82115 | 0.024196 |  | Tcf15 | –1.00965 | 1.49E–12 |
| Lgals3bp | –0.62544 | 1.84E–14 |  | Tmem88 | –0.80065 | 1.14E–12 |
| Lin9 | –0.62486 | 0.000165 |  | Tomm7 | –0.79847 | 2.86E–07 |
| Ltc4s | –1.10115 | 0.00606 |  | Usmg5 | –1.49256 | 0.010782 |
| Magohb | –0.61692 | 0.000599 |  | Vtn | –0.64253 | 4.85E–22 |
| Maob | –0.76571 | 2.23E–11 |  | Mlf1 | –0.72835 | 5.41E–18 |
| Mlf1 | –0.72835 | 5.41E–18 |  | Mrpl33 | –6.523 | 1.93E–09 |
